# Simultaneous silencing of ACSL4 and induction of GADD45B in hepatocellular carcinoma cells amplifies the synergistic therapeutic effect of aspirin and sorafenib

**DOI:** 10.1038/cddiscovery.2017.58

**Published:** 2017-09-11

**Authors:** Hongping Xia, Kee Wah Lee, Jianxiang Chen, Shik Nie Kong, Karthik Sekar, Amudha Deivasigamani, Veerabrahma Pratap Seshachalam, Brian Kim Poh Goh, London Lucien Ooi, Kam M Hui

**Affiliations:** 1 Laboratory of Cancer Genomics, National Cancer Centre, Singapore, Singapore; 2 Department of Pathology, School of Basic Medical Sciences & Sir Run Run Hospital, Nanjing Medical University, Nanjing, China; 3 Department of General Surgery, Singapore General Hospital, Singapore, Singapore; 4 Department of Surgical Oncology, National Cancer Centre, Singapore, Singapore; 5 Cancer and Stem Cell Biology Program, Duke-NUS Medical School, Singapore, Singapore; 6 Institute of Molecular and Cell Biology, A*STAR, Biopolis Drive Proteos, Singapore, Singapore; 7 Department of Biochemistry, Yong Loo Lin School of Medicine, National University of Singapore, Singapore, Singapore

## Abstract

Sorafenib is currently the only US Food and Drug Administration (FDA)-approved molecular inhibitor for the systemic therapy of advanced hepatocellular carcinoma (HCC). Aspirin has been studied extensively as an anti-inflammation, cancer preventive and therapeutic agent. However, the potential synergistic therapeutic effects of sorafenib and aspirin on advanced HCC treatment have not been well studied. Drug combination studies and their synergy quantification were performed using the combination index method of Chou-Talalay. The synergistic therapeutic effects of sorafenib and aspirin were evaluated using an orthotopic mouse model of HCC and comprehensive gene profiling analyses were conducted to identify key factors mediating the synergistic therapeutic effects of sorafenib and aspirin. Sorafenib was determined to act synergistically on HCC cells with aspirin *in vitro*. Using Hep3B and HuH7 HCC cells, it was demonstrated that sorafenib and aspirin acted synergistically to induce apoptosis. Mechanistic studies demonstrated that combining sorafenib and aspirin yielded significant synergistically anti-tumor effects by simultaneously silencing ACSL4 and the induction of GADD45B expression in HCC cells both *in vitro* and in the orthotopic HCC xenograft mouse model. Importantly, clinical evidence has independently corroborated that survival of HCC patients expressing ACSL4^high^GADD45B^low^ was significantly poorer compared to patients with ACSL4^low^GADD45B^high^, thus demonstrating the potential clinical value of combining aspirin and sorafenib for HCC patients expressing ACSL4^high^GADD45B^low^. In conclusion, sorafenib and aspirin provide synergistic therapeutic effects on HCC cells that are achieved through simultaneous silencing of ACSL4 and induction of GADD45B expression. Targeting HCC with ACSL4^high^GADD45B^low^ expression with aspirin and sorafenib could provide potential synergistic therapeutic benefits.

## Introduction

Hepatocellular carcinoma (HCC) is the second leading cause of cancer-related deaths worldwide and the global incidence is rising. An estimated 782 500 new liver cancer cases and 745 500 deaths occurred worldwide during 2012, with China alone accounting for about 50% of the total number of cases and deaths.^1,2^ Unfortunately, most HCC patients are still being diagnosed in a late stage and the only treatment available to these patients is sorafenib.^3^ Although the data from the Sorafenib Hepatocellular Carcinoma Assessment Randomized Protocol (SHARP) trial^4^ and the Asia-Pacific study^5^ could demonstrate a significant survival benefit, the absolute gain in life expectancy was marginal (2–3 months overall survival benefit compared with placebo) and hence, the cost effectiveness of sorafenib is a big concern. Recent clinical studies with immune checkpoint inhibitors have emerged as promising therapeutic strategies for HCC; nevertheless, these studies are preliminary and the treatment of advanced or metastatic HCC currently remains a high unmet medical need.^6^

The combination of two or more network-targeted drugs with the aim of decreasing the dosage and toxicities of a single drug to enhance therapeutic responses is an established approach to treat many diseases like acquired immunodeficiency syndrome, complex infections, hematologic malignancies and solid tumors.^7^ The non-steroidal anti-inflammatory drug (NSAID) aspirin is one of the cheapest and most common drugs on the market. Aspirin has been used as an analgesic/anti-inflammatory drug and in the prevention of cardiovascular disease. It has recently been shown to protect against certain types of cancer.^8^ Being a clear example of inflammation-driven cancer,^9^ we have explored the possibility of aspirin to enhance the therapeutic effects of sorafenib in a preclinical model of advanced orthotopic HCC.

## Results

### Synergistic inhibitory effects of combining aspirin and sorafenib on liver cancer cells

**Figure 1:**
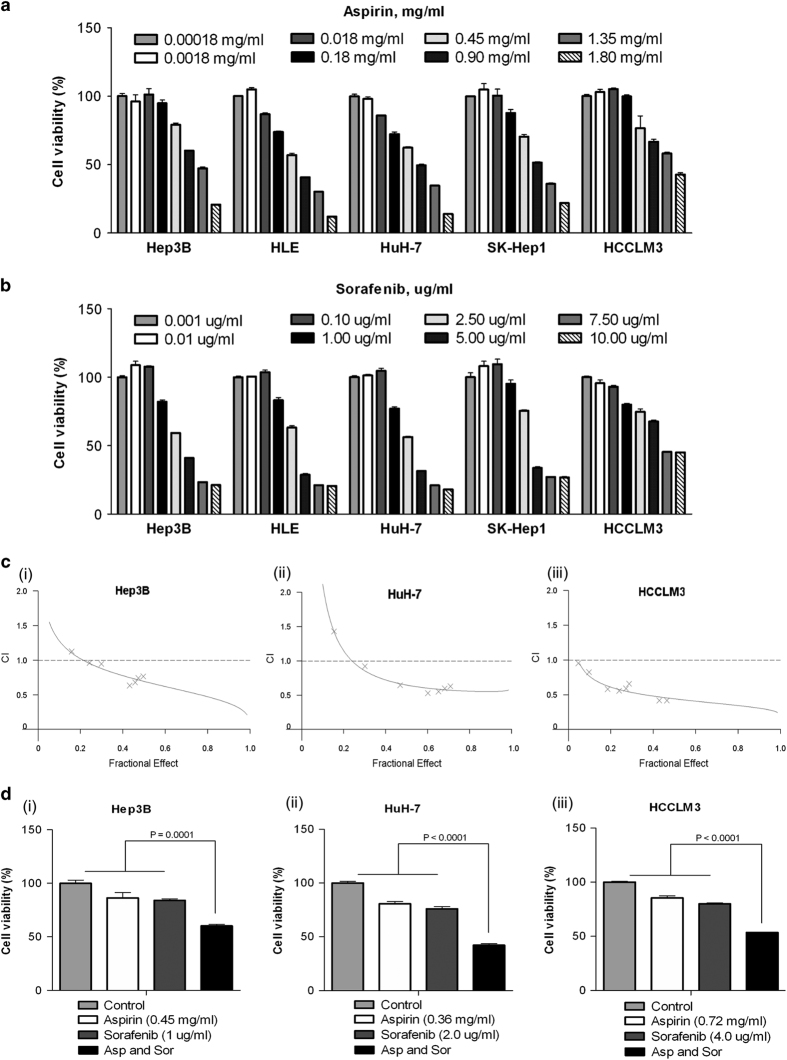
The inhibitory effects of aspirin and sorafenib and synergistic inhibition effects of combined aspirin and sorafenib in different liver cancer cells *in vitro*. (**a** and **b**) Dose-escalation effects of aspirin (**a**) and sorafenib (**b**) on cell viability in five HCC cell lines was determined with MTS assays. (**c**) Synergistic curve of interactions between aspirin and sorafenib in Hep3B (i), HuH-7 (ii) and HCCLM3 (iii) cells. Cells were treated with aspirin or sorafenib either individually or in combination over a range of concentrations at a fixed ratio for 24 h. After the cell viability was determined with MTS assays under each condition, the combination index was calculated as described in the Materials and Methods. CI values of less than one are considered synergism. (**d**) Combined effects of aspirin and sorafenib in Hep3B (i), HuH-7 (ii) and HCCLM3 (iii) cells. Cells were exposed to study agents either individually or in combination at indicated concentrations for 24 h and cell viability was assessed using MTS assays. All data were plotted as the percentage of the control (DMEM) treated cells. Points, mean; bars, S.E. (*n*=3).

To investigate the anticancer therapeutic effects of aspirin and sorafenib, we first employed a panel of liver cancer cell lines including Hep3B, HLE, HuH7, SK-HEP-1 and HCCLM3 to assess their growth inhibition response to aspirin and sorafenib treatment *in vitro*. As shown in Figures 1a and b, the growth inhibitory effects of aspirin and sorafenib on the HCC cells were variable but in a dose-dependent fashion. The concentrations of aspirin and sorafenib needed to inhibit growth by 50% (IC50) for the HCC cell lines were 1.5 mg/ml and 4.7 *μ*g/ml for aspirin and sorafenib, respectively (Supplementary Table S1). To investigate the potential synergistic therapeutic benefits of combining aspirin and sorafenib, the HCC cell lines Hep3B, HuH-7 and HCCLM3, which exhibited different sensitivity to aspirin and sorafenib (Figures 1a and b) were selected for further study and the synergistic effect was evaluated over a range of concentrations. The dose-dependent inhibition of cell growth for all three HCC cell lines studied was significantly enhanced when they were treated with aspirin and sorafenib and subsequent assessment of the synergistic interactions by CalcuSyn analysis^10^ yielded combination index (CI) values of mostly <1, thus indicating that the therapeutic effect of combining aspirin and sorafenib on HCC cells was indeed synergistic (Supplementary Table S2).

To ascertain the strongest synergistic combinations, we tested different ratios and concentrations of drug combinations on the HCC cells and demonstrated that the best CI of 0.634 was obtained at 0.45 mg/ml aspirin and 1.0 *μ*g/ml sorafenib for Hep3B cells, the CI of 0.533 was obtained at 0.36 mg/ml aspirin and 2.0 *μ*g/ml sorafenib for HuH-7 cells and the CI of 0.419 was obtained at 0.72 mg/ml aspirin and 4.0 *μ*g/ml sorafenib for HCCLM3 cells (Supplementary Table S2 and Figure 1c). For validation, the concentration of drug that gave the highest CI for the respective HCC cell line was tested again both in combination and individually. Subsequent ANOVA analysis demonstrated that the synergistic growth inhibition effect obtained with aspirin and sorafenib was significantly different from individual drug treatment (Figure 1d). The aspirin and sorafenib drug concentrations that gave the strongest synergistic growth inhibition for Hep3B, HuH-7 and HCCLM3 were selected for subsequent analysis, unless otherwise indicated.

### Combinatorial treatment of HCC cells with aspirin and sorafenib increased apoptosis

**Figure 2:**
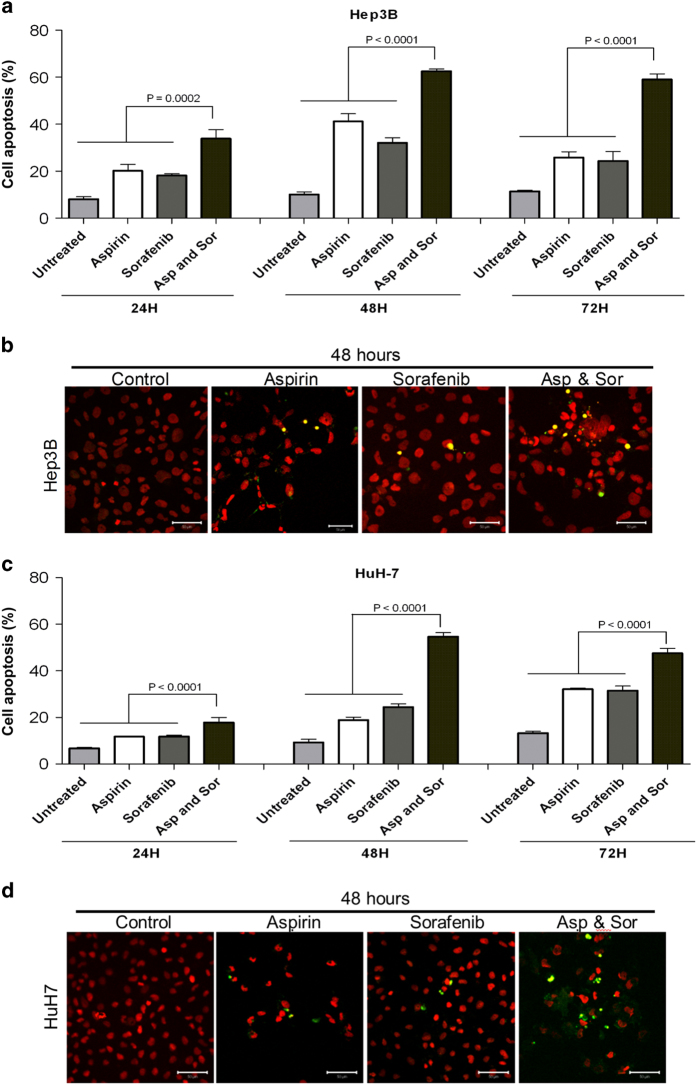
The synergistic inhibition effects of combination aspirin and sorafenib in HCC cells. Aspirin- and sorafenib-induced apoptosis and DNA fragmentation in Hep3B and HuH-7 cells, respectively. (**a** and **c**) Quantification analysis of total cell death from flow cytometry. Annexin V-PE/7-AAD analysis of Hep3B (**a**) and HuH-7 (**c**) cells after treated for 24, 48 and 72 h with study agents. Floating and adherent cells were collected after treatment and analyzed by flow cytometry. Points, mean; bars, S.E. (*n*=3). (**b** and **d**) TUNEL staining of Hep3B (**b**) and HuH-7 (**d**) cells after 48 h of treatment with study agents. The bright yellow fluorescence spots indicated the apoptotic cells. Nuclei were counterstained with 7-AAD. TUNEL-stained cells were observed at ×40 magnification and scale bars indicate 50 *μ*m.

The ability of aspirin and sorafenib, both in isolation and in combination, to induce apoptosis in Hep3B and HuH-7 cells was compared after treatment for 24, 48 and 72 h. Aspirin and sorafenib significantly enhanced apoptosis compared to aspirin or sorafenib. The apoptosis was 62.6±0.9% in Hep3B cells (Figure 2a and Supplementary Figure S1A) and 54.7±1.7% in HuH-7 cells (Figure 2c and Supplementary Figure S1B) at 48 h. TUNEL staining also demonstrated increased percentages of apoptotic HCC cells following treatments with aspirin and sorafenib compared to treatments with either aspirin or sorafenib (*P*<0.05, Figures 2b and d).

### Transcriptome analysis revealed that ACSL4 and GADD45B mediated the synergistic effects of aspirin and sorafenib-treated HCC cells

**Figure 3:**
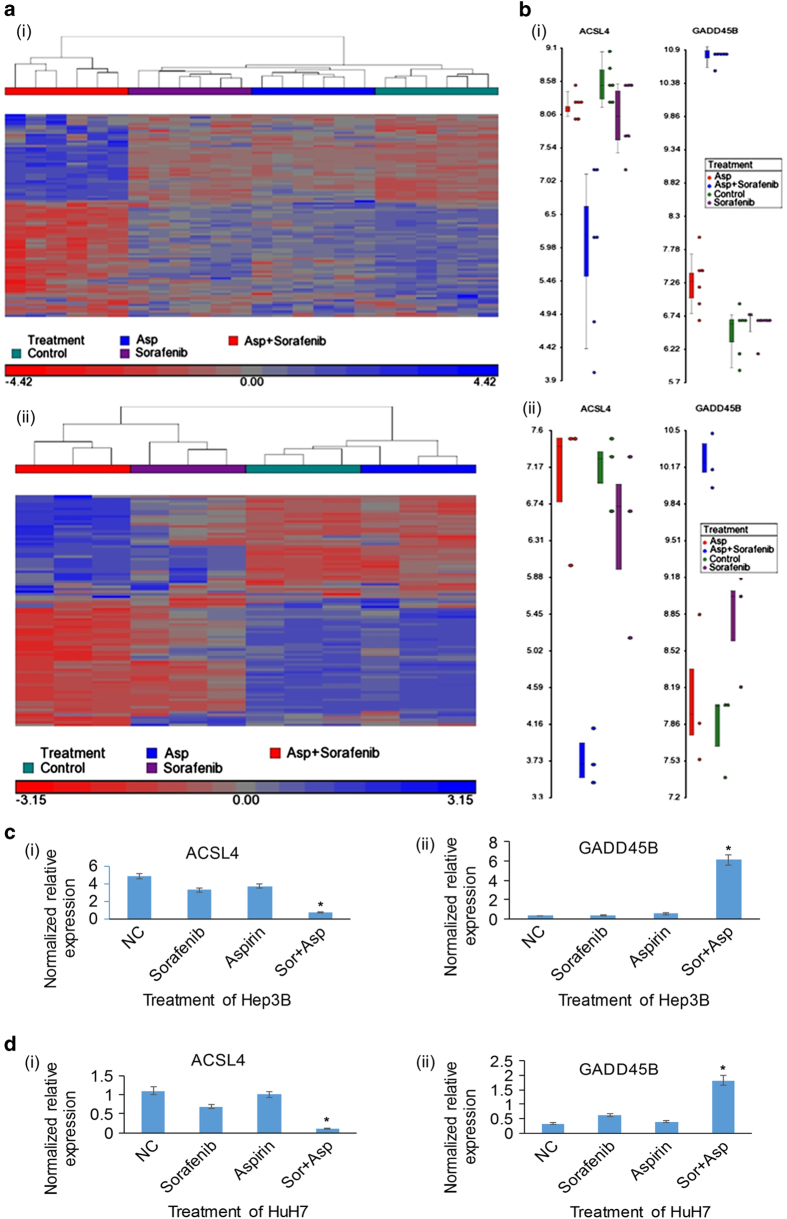
Transcriptome analysis of HCC cells in response to aspirin and sorafenib. (**a**) The heatmap shows a panel of HCC dysregulated genes significantly enriched in combination treatment of aspirin and sorafenib in Hep3B (i) and HuH7 (ii) cells. (**b**) The dot plot showed the synergistic blockade of ACSL4 and induction of GADD45B by aspirin and sorafenib combination in Hep3B (i) and HuH7 (ii) cells by microarray analysis. (**c** and **d**) The real-time RT-qPCR analysis demonstrated a synergistic blockade of ACSL4 (i) and induction of GADD45B (ii) by the aspirin and sorafenib combination but not in single drug treatment in Hep3B (**c**) and HuH7 (**d**) cells. *P<0.05.

To decipher the underlying molecular mechanisms mediating the synergistic therapeutic effects observed with aspirin and sorafenib, we proceeded to identify genes that were significantly enriched when either Hep3B cells or HuH-7 cells were treated with aspirin and sorafenib individually or in combination using Affymetrix Human Genome U133 plus 2.0 Arrays, followed by bioinformatic analysis using the Partek Genomics Suite software. These genes were then compared with the gene expression data set that we have previously established with human HCC tumors and adjacent histologically normal liver tissues.^11,12^ Bioinformatic analysis revealed a set of significantly dysregulated genes that were upregulated in HCC human tissues but downregulated by treatment with aspirin and sorafenib (Figure 3a, Supplementary Table S3). These dysregulated genes are mainly associated with cell cycle control, response to DNA damage and apoptosis-related pathways (Supplementary Figures S2A and B). Among them, the expression of ACSL4 was the most significantly downregulated while the expression of GADD45B was the most significantly ameliorated by treatment with aspirin and sorafenib for both Hep3B and HuH-7 cells (Figure 3b). Real-time RT-qPCR analysis also confirmed the inhibition of ACSL4 expression and amelioration of GADD45B expression following treatment with aspirin and sorafenib but not with aspirin or sorafenib individually in both Hep3B cells (Figure 3c) and HuH-7 cells (Figure 3d).

**Figure 4:**
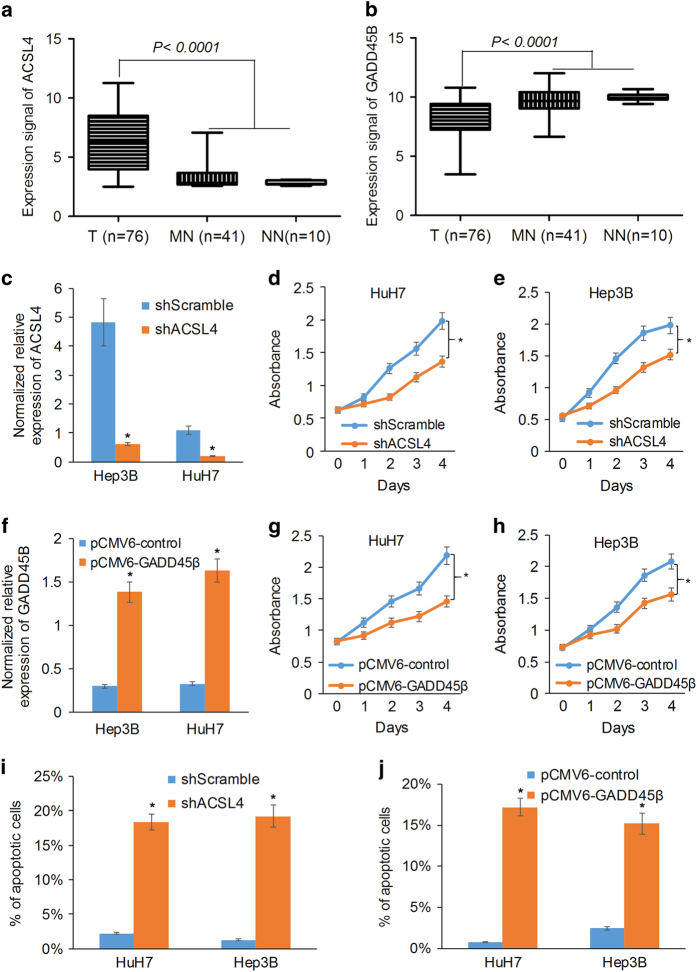
The critical role of ACSL4 and GADD45B in HCC cell growth and apoptosis. (**a** and **b**) The box plot shows that the expression of ACSL4 (**a**) was significantly upregulated while the expression of GADD45B (**b**) was significantly downregulated in most HCC tumors in our established global gene expression database on human HCC tumors (T) and matched normal (MN) or histologically normal liver tissues (NN). (**c**) The normalized relative expression level of ACSL4 in HCC cells transfected with the short hairpin RNA (shRNA) of ACSL4 and Scramble control. (**d** and **e**) The cell growth curve showed that knockdown of ACSL4 significantly inhibits HCC cell growth. (**f**) The normalized relative expression level of GADD45B in HCC cell transfected with pCMV6-GADD45B plasmid. (**g** and **h**) The cell growth curve showed that overexpression of GADD45B significantly inhibits HCC cell growth. (**i** and **j**) Knockdown of ACSL4 or overexpression of GADD45B significantly induces apoptosis of HCC cells with an increasing Annexin V^+^ cell population through Annexin V-PE/7-AAD analysis by flow cytometry. *P<0.05.

The expression of ACSL4 was significantly upregulated while the expression of GADD45B was significantly downregulated in the HCC tumor tissues compared to matched normal tissues or histologically normal liver tissues from colorectal cancer patients who had liver metastases (Figures 4a and b). Knockdown of ACSL4 expression with the short hairpin RNA (shRNA) (Figure 4c) or overexpression of GADD45B by transfecting pCMV6-GADD45B plasmid (Figure 4f) into Hep3B and HuH7 cells significantly inhibited cell growth (Figures 4d, e, g and h) and induced the apoptosis of HuH7 and Hep3B cells as suggested by the increase of Annexin V-positive cells (Figures 4i and j).

Furthermore, ACSL4^+^GADD45B^−^ and ACSL4^−^GADD45B^+^ cells were purified from Hep3B and HuH-7 cells using flow cytometry. Cell viability assays demonstrated that the combinatorial growth inhibitory effect of aspirin and sorafenib was significantly more potent on the purified ACSL4^+^GADD45B^−^ cells than on the ACSL4^−^GADD45B^+^ cells (Supplementary Figure S3A). Annexin V-PE/7-AAD analysis also showed that aspirin and sorafenib-induced apoptosis was more effective for the ACSL4^+^GADD45B^−^ cells than for the ACSL4^−^GADD45B^+^ cells (Supplementary Figures S3B and C), thus suggesting ACSL4 and GADD45B may play critical regulatory roles in mediating the synergistic therapeutic effect of aspirin and sorafenib in HCC cells.

### Combinatorial treatment with aspirin and sorafenib significantly reduced tumor growth in the orthotopic HCC xenograft mouse model

**Figure 5:**
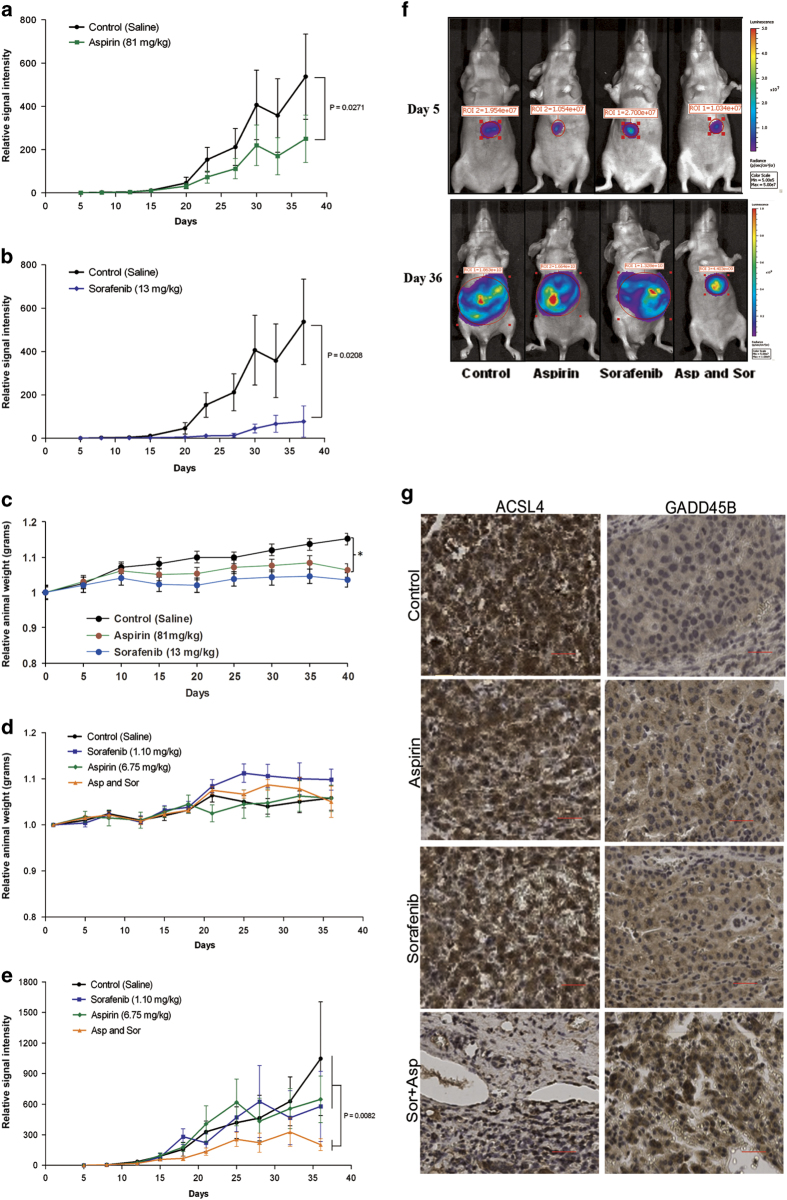
The effect of combination therapy on HuH-7-Luc2 xenograft in nude mice. (**a** and **b**) Antitumor effects of aspirin or sorafenib at the clinical recommended dose. Nude mice were treated with daily oral gavage of either aspirin (81 mg/kg) or sorafenib (13 mg/kg b.i.d.). Points, mean; bars, S.E. (*n*=6). (**c**) Relative body weight change of the tumor-bearing mice during the treatment. The significant difference between all treatment groups and control group was observed (*P<0.05). (**d**) Relative body weight change of the tumor-bearing mice during the treatment. No significant difference was found between any treatment group and the control group. Points represent mean; bars, S.E. (*n*=5). (**e**) The combination of aspirin and sorafenib shows significant antitumor effects on HuH-7-Luc2 tumors. Mice received solvent control, aspirin, sorafenib, or a combination of aspirin plus sorafenib as described in the Materials and Methods. Graph showing relative tumor burden at different times during the course of the experiment. Points represent mean; bars, S.E. (*n*=5). (**f**) The representative images for bioluminescence imaging luciferase signal of tumorbearing mice in different treatment groups at d5 and d36. (**g**) The representative IHC images of ACSL4 and GADD45B staining in the tumors of different treatment groups.

To determine the potential clinical utility of combinatorial treatment of aspirin and sorafenib for HCC, we evaluated the anti-tumor effects of combining aspirin and sorafenib with an orthotopic HCC xenograft mouse model. First, the normal recommended daily dose of 81 mg/kg for aspirin and 13 mg/kg bid for sorafenib was employed. At this dose, both aspirin and sorafenib significantly inhibited the growth of HCC xenograft tumor individually (Figures 5a and b) but with observable toxicity and significant body weight loss (Figure 5c). The potential toxicities observed with prolonged treatment with high doses of aspirin and sorafenib *in vivo* prompted us to reduce the doses of aspirin and sorafenib for the combined therapy. To maximize the observable therapeutic combined effects and minimize the potential side-effects in mice, we made stepwise dosage adjustments of aspirin and sorafenib and finally adjusted the combined dose to 6.75 mg/kg aspirin and 1.1 mg/kg bid sorafenib. At this dose, no toxicity and abnormal body weight loss were observed with the orthotopic HuH-7-Luc2 tumor model (Figure 5d). Moreover, results showed that the observed therapeutic effect following treatments with aspirin or sorafenib individually at this lower dose was only marginal (Figure 5e). In comparison, tumor growth was significantly inhibited by treatments with the combination of aspirin and sorafenib (Figures 5e and f). Compared to other treatment groups, immune-histochemical studies of tumor tissue samples from the group treated with aspirin and sorafenib further indicated that the expression of ACSL4 was significantly decreased while the expression of GADD45B was significantly increased (Figure 5g), thus supporting the observations made with the *in vitro* and mechanistic studies earlier.

### Potential application of ACSL4 and GADD45B as companion biomarkers towards the goal of precision treatment for HCC

**Figure 6:**
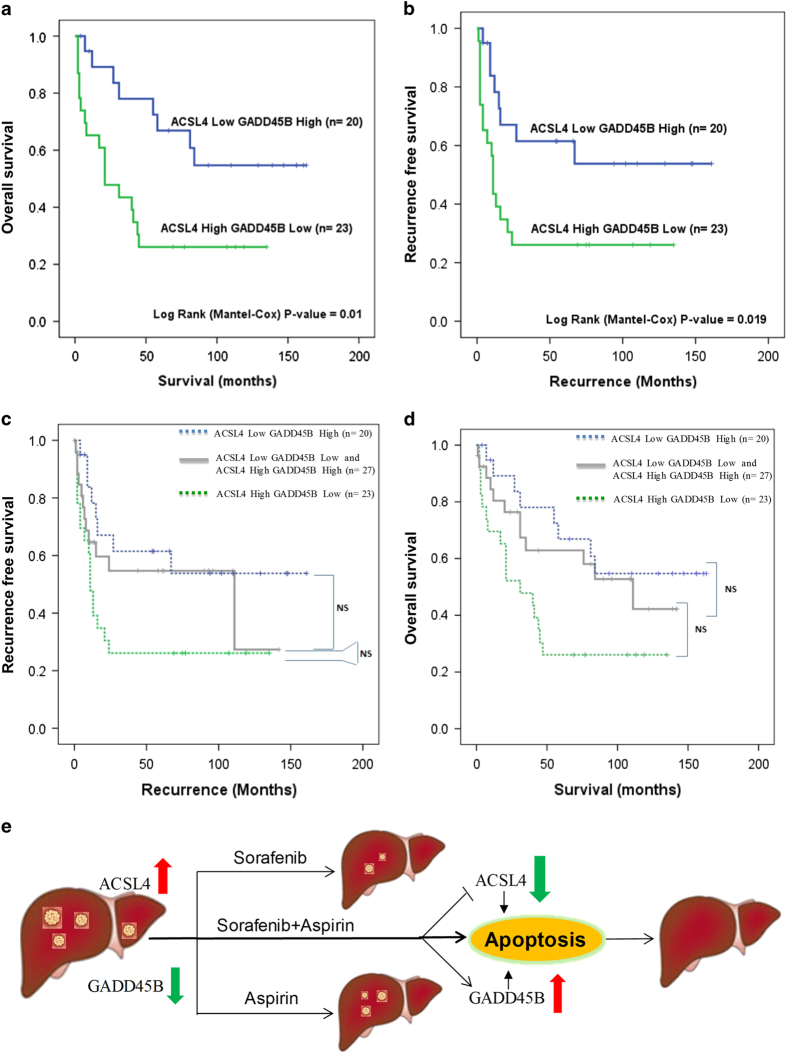
Combination of aspirin and sorefenib is optimized to treatment of advanced HCC patients with high ACSL4 and low GADD45B expression. (**a** and **b**) ACSL4 high and GADD45B low in HCC tissues were significantly associated with poor overall survival (**a**) and recurrence-free survival (**b**) in our data set. The median expression value obtained for ACSL4 and GADD45B in the samples studied was chosen as the cut-off point for survival analysis using the Kaplan–Meier method. (**c** and **d**) The other HCC patients that are either low ACSL4 and low GADD45B or high ACSL4 and high GADD45B, the difference in survival was in between. (**e**) A schematic diagram has been put forward to summarize that silenced ACSL4 and induced GADD45B expression by aspirin and sorafenib combination is promising to treat liver cancer.

Previously, we have established an expression data set of human HCC tumor with adjacent histologically normal liver tissues and the survival information of these patients.^11,12^ Of the 43 HCC patients analyzed that either expressed low ACSL4 and high GADD45B or high ACSL4 and low GADD45B, we observed a significant difference in the overall and recurrence-free survival of these two groups of patients (Figures 6a and b) using the median expression value of ACSL4 and GADD45B as the cut-off for high and low ACSL4 and GADD45B expression, respectively. When compared to the other HCC patients that have either low ACSL4 and low GADD45B or high ACSL4 and high GADD45B, the difference in survival was in between (Figures 6c and d). The observed difference between the overall survival and recurrence-free survival of HCC patients with single high ACSL4 or low ACSL4 was not significant, while single high GADD45B and low GADD45B was marginal (Supplementary Figures S4A–D). We have demonstrated earlier that the combinatorial therapy with aspirin and sorafenib decreased the expression of ACSL4 and increased the expression of GADD45B to result in the inhibition of tumor growth. It would therefore imply that the clinical utility of the combinatorial aspirin and sorefenib would potentially give the best therapeutic effects for advanced HCC patients with high ACSL4 and low GADD45B expression (Figure 6e).

## Discussion

For patients with unresectable or metastatic HCC, conventional chemotherapy is of limited benefit. Sorafenib, a multi-kinase inhibitor, is the only drug that has been approved by the US FDA for the treatment of advanced HCC, which has demonstrated a statistically significant, but only modest, overall survival benefit. Moreover, drug resistance develops in many different tumor types, including HCC. The challenges to develop systemic targeted treatment for HCC are manifold. One of the main hurdles is to identify clinically relevant and applicable molecular treatment stratification for HCC. Although many clinical trials of novel targeted drugs and combinatorial therapies have been carried out with many more in progress, patients with advanced HCC still have a dismal prognosis.

Recently, there is growing evidence to suggest that aspirin, an NSAID often used to treat symptoms aggravated by inflammation, can have significant therapeutic effects against many human cancers. It has been reported that patients with stage III colon cancer who used aspirin had a 29% improvement in survival outcomes and patients who initiated the use of aspirin obtained a 47% improvement in mortality outcomes.^13^ Long-term follow-up of randomized trials of aspirin in prevention of vascular events showed that daily aspirin reduced the incidence of colorectal cancer and several other cancers and reduced metastasis.^14^ HCC is an example of inflammation-related cancer and a chronic inflammatory state appears to be necessary for its initiation and development. The chronic inflammation is associated with the continued expression of cytokines and recruitment of immune cells to the liver. Activated inflammatory cells release free radicals, such as reactive oxygen species and nitric oxide reactive species, which in turn can cause DNA damage and lead to gene mutations, thus fostering neoplastic transformation.^15,16^ The chronic inflammation also affects many cellular pathways, leading to fibrosis and cirrhosis and finally hepatocarcinogenesis. Aspirin can reduce oxidative stress, protect against oxidative damage and may minimize the pro-metastasis effect of sorafenib by upregulating the expression of HTATIP2 and downregulating SDF1-*α* expression in the tumor microenvironment of HCC.^17,18^ However, we observed that the expression of HTATIP2 was not significantly modulated in HCC in our data set (Supplementary Figure S5A), TCGA (Supplementary Figure S5C) and GSE14520 data set (Supplementary Figure S5E), while the expression of SDF1-*α* (CXCL12) was significantly decreased in HCC (our data set – Supplementary Figure S5B, TCGA-S5D and GSE14520-S5F), suggesting that the synergistically anti-tumor effect and mechanism of aspirin and sorafenib in advanced HCC is still mostly unclear.

In this study, we have systematically demonstrated the synergistic therapeutic effects of aspirin and sorafenib in HCC cells and with the orthotopic HCC mouse model for HCC. Comprehensive gene profiling and bioinformatic analysis identified ACSL4 and GADD45B as the key molecules mediating the synergistic therapeutic effects of aspirin and sorafenib. ACSL4 (or FACL4) is a central enzyme controlling the unesterified arachidonic acid level in cells.^19^ The expression of ACSL4 was consistently shown to be upregulated in most of the HCC patient samples in our previously published expression data set^11,12,20^ and other published data sets, including TCGA (Supplementary Figure S6A) and GSE14520 (Supplementary Figure S6B), respectively. Knockdown of ACSL4 significantly inhibited HCC cell growth and induced apoptosis, suggesting ACSL4 may serve as a potential novel therapeutic target for HCC. GADD45B belongs to a family of small-molecule proteins that play important roles in regulating cellular stress response, survival, senescence and apoptosis. The expression of GADD45B is consistently shown to be downregulated in most of the HCC patient samples studied in TCGA (Supplementary Figure S6C) and GSE14520 (Supplementary Figure S6D) and the induction of GADD45B expression contributed to sorafenib-induced apoptosis in HCC cells.^21^

HCC patients with high ACSL4 and low GADD45B expression have a much poorer overall and recurrence-free survival compared to patients with low ACSL4 and high GADD45B expression (Figures 6a and b). In this study, we have demonstrated that combinatorial therapies with aspirin and sorafenib targeted the apoptosis-related pathways mediated by ACSL4 and GADD45B by silencing ACSL4 and inducing GADD45B expression (Figure 6e). Therefore, the expression of ACSL4 and GADD45B on HCC patients’ samples can potentially act as companion diagnostic biomarkers to facilitate the selection of patients with high ACSL4 and low GADD45B expression for treatment with aspirin and sorafenib.

In conclusion, we demonstrate significant synergistic anti-tumor effects by combining sorafenib and aspirin. Mechanistically, the observed synergistic anti-tumor effect is mediated through the simultaneously silencing of ACSL4 and induction of GADD45B expression in HCC cells. Importantly, HCC patients with ACSL4^high^GADD45B^low^ have significantly worse prognoses than patients with ACSL4^low^GADD45B^high^. Therefore, HCC patients with ACSL4^high^GADD45B^low^ expression could be predicted to benefit from the combination therapy with aspirin and sorafenib.

## Materials and methods

### Cell lines and cell culture

The HuH7 and HLE cells were bought from the Japanese Collection of Research Bioresources (JCRB) Cell Bank (Osaka, Japan) and the Hep3B and SK-HEP-1 cells were bought from American Type Culture Collection (ATCC) (Manassas, VA, USA). HCCLM3 was a kind gift from Professor Zhao-You Tang at the Liver Cancer Institute (Zhongshan Hospital, Fudan University, Shanghai, China). The cell lines HLE, Hep3B and SK-HEP-1 have been authenticated using the short tandem repeat profiling authentication test by Bio-Synthesis Inc (Lewisville, TX, USA) while HuH7 has been tested by Genetica DNA Laboratories (Cincinnati, OH, USA). The cells were cultured in Dulbecco’s modified Eagle’s medium (DMEM) with 10% FBS and 100 units/ml of penicillin and 100 *μ*g/ml of streptomycin (Invitrogen, Carlsbad, CA, USA) at 37 °C in the presence of 5% CO_2_.

### Animal study in the orthotopic HCC model

Female BALB/c nude mice (7 weeks of age) were obtained from BioLasco (Taipei, Taiwan). All procedures involving animals were reviewed and approved by the SingHealth Institutional Animal Use and Care Committee. HuH-7-Luc2 tumor cubes were inoculated subcutaneously into the liver of the mice. Mice were randomized into each experimental and control groups 14 days after surgery to start the treatment with a similar tumor burden in each group. To determine the effect of aspirin and sorafenib on tumor growth, mice were treated with daily oral gavage of either (1) one dose of aspirin (81.00 mg/kg), (2) two doses of sorafenib (13 mg/kg), or (3) one dose of saline as control. For *in vivo* drug combination experiments, tumor-bearing mice were given daily oral injections of either (1) one dose of aspirin (6.75 mg/kg), (2) two doses of sorafenib (1.1 mg/kg), (3) the combination of aspirin and sorafenib, or (4) a single dose of saline as a control. The tumor burden in response to different treatments was monitored by bioluminescence imaging on the Xenogen IVIS system (Caliper Life Science, Perkin-Elmer Company, Hopkinton, MA, USA). To assess treatment-related toxicities, treated and untreated tumor-bearing mice were weighed before and regularly after treatments.

### Cell viability assay

Cells were seeded in a 96-well plate at a density of ~6×10^3^ cells per well. One day after seeding, the culture medium was replaced with fresh DMEM containing aspirin or sorafenib at indicated concentration and incubated for 24 h. Cell viability was assessed using the CellTiter 96 AQueous One Solution Cell Proliferation Assay kit (MTS assay, G3581, Promega Corporation, Madison, WI, USA) according to the manufacturer’s instructions.

### Determination of synergism

The potential of drug synergism was determined using the method of Chou and Talalay implemented in the software package CalcuSyn (Biosoft, Cambridge, UK). A CI of less than one indicates synergy.

### Cell apoptosis assay

Annexin V-PE/7-AAD (BD Biosciences, San Jose, CA, USA) and terminal deoxynucleotidyl transferase-mediated nick end labeling (TUNEL, Promega Corporation) assays were used to measure the percentage of drug-induced apoptotic cell death. Cells were seeded in 60 mm culture dishes or six-well plates at a density of ~1×10^6^ cells per well and treated with aspirin, sorafenib or a combination at the indicated concentration. Cells treated with DMEM were used as the control group. Floating and adherent cells were collected after 24, 48 and 72 h of treatment. The annexin V-PE/7-AAD stained cells were analyzed using flow cytometry (BD Biosciences), while TUNEL assay were only performed on cells after 48 h of treatment and stained cells were analyzed by confocal microscopy (Carl Zeiss).

### Isolation of total RNA and Affymetrix GeneChip experiments

Gene profiling analyses following the 24 h treatment with aspirin, sorafenib or a combination were done with Affymetrix Human Genome U133 Plus 2.0 Arrays according to the manufacturer’s protocol. Briefly, total RNA was isolated using TRIzol reagent (Life Technologies, Carlsbad, CA, USA). RNA yield and quality was assessed using a NanoDrop Spectrophotometer (Thermo Scientific, Waltham, MA, USA) and an Agilent 2100 bioanalyzer (Agilent Technologies, Santa Clara, CA, USA), respectively. Total extracted RNA (500 ng) was reverse transcribed and biotin labeled. The final cRNA obtained was used to hybridize to the GeneChip and scanned on a GeneChip Scanner (Affymetrix, Santa Clara, CA, USA). Signal intensities were transformed to log2 base and imported to Partek Genomics Suite software (Partek Inc., St Louis, MO, USA) to conduct statistical analyses.

### Fluorescence activated cell sorting

For isolation of the ACSL4^+^GADD45B^−^ or ACSL4^−^GADD45B^+^ cell populations from Hep3B and HuH7 cells, cells were fixed with 2% paraformaldehyde and were stained with Anti-FACL4 antibody [EPR8640] (Alexa Fluor 647) and Anti-GADD45B antibody (FITC) (Abcam, Cambridge, MA, USA), or isotype-control. The samples were washed twice and analyzed or sorted on a BD FACSAria (BD Biosciences).

### HCC samples and reagents

The collection of tumor and adjacent normal liver tissues from HCC patients was approved by our Institutional Review Board (IRB) and all tissues studied were provided by the Tissue Repository of the National Cancer Centre Singapore (NCCS). Aspirin and sorafenib (Nexavar) were kindly provided by Sigma-Aldrich Chemical (St Louis, MO, USA) and Bayer HealthCare Pharmaceuticals (Whippany, NJ, USA), respectively. Both compounds were dissolved in 100% DMSO and diluted to appropriate concentrations with DMEM for *in vitro* studies or with saline for *in vivo* studies.

### Quantitative real-time PCR

Extracted total RNA was reverse transcribed into single-stranded cDNA using a Reverse Transcription Supermix (Bio-Rad, Hercules, CA, USA). Real-time PCR amplification was carried out with SsoFast EvaGreen Supermix (Bio-Rad) using a Bio-Rad Real-Time PCR System under the following conditions: 95 °C for 30 s, 40 cycles of 95 °C for 5 s, and 58 °C for 5 s. All oligonucleotide primers for PCR were chemically synthesized by AIT biotech. Housekeeping genes 18S ribosomal RNA or HPRT1 were used to normalize the expression levels in the subsequent quantitative analyses.

### Functional analysis using the ingenuity pathways system

Network visualization and functional analysis was done using IPA in Ingenuity System. Data sets containing gene identifiers of each treatment group were uploaded into the web-based application and each gene identifier was mapped to its corresponding gene object in the Ingenuity Pathways Knowledge Base. Networks were generated and ranked according to a score calculated via Fisher’s exact test. The functional analysis of a network identified the biological functions and/or diseases that were most significant to the genes in the network. The network genes associated with biological functions and/or diseases were considered for further analysis.

### Immunohistochemistry

The paraffin-embedded tissue samples from consenting patients were cut in 5 *μ*m sections and placed on polylysine coated slides, deparaffinized in xylene and rehydrated using a series of graded alcohols. Antigen retrieval was performed by heat mediation in citrate buffer (pH 6) (Dako, Carpinteria, CA, USA). Samples were blocked with 10% goat serum before incubating with primary antibody. The samples were incubated overnight with primary antibody: anti-ACSL4 and GADD45B (1 : 100) (Abcam) or an isotype-matched IgG as a negative control in a humidified container at 4 °C. Immunohistochemical staining was performed with the Dako Envision Plus System (Dako) according to the manufacturer’s instructions.

### Statistical analysis

Data points are presented as mean±S.D. Differences between groups within experiments were analyzed in GraphPad Prism 5.0 software (GraphPad) using independent samples *t*-test or analysis of variance (ANOVA). All statistical significance was set at *P*⩽0.05.

## Additional information

**Publisher’s note:** Springer Nature remains neutral with regard to jurisdictional claims in published maps and institutional affiliations.
