# Supplementary material for: Simultaneous silencing of ACSL4 and induction of GADD45B in hepatocellular carcinoma cells amplifies the synergistic therapeutic effect of aspirin and sorafenib
Source: Cell Death Discov. 2017 Sep 11;3:17058–. doi: 10.1038/cddiscovery.2017.58 (PMC5592242; doi:10.1038/cddiscovery.2017.58)

**Supplementary Data**

**Simultaneous silencing of ACSL4 and induction of GADD45B in hepatocellular carcinoma cells amplify the synergistic therapeutic effect of aspirin and sorafenib**

Hongping Xia^1, 2.*,#^, Kee Wah Lee^1,*^ , Jianxiang Chen^1^, Shik Nie Kong^1^, Karthik Sekar^1^, Amudha Deivasigamani^1^, Veerabrahma Pratap Seshachalam^1^, Brian Kim Poh Goh^4^, London Lucien Ooi ^4,5^, Kam M. Hui^1,2,3,6,7 #^

**Supplementary Tables**


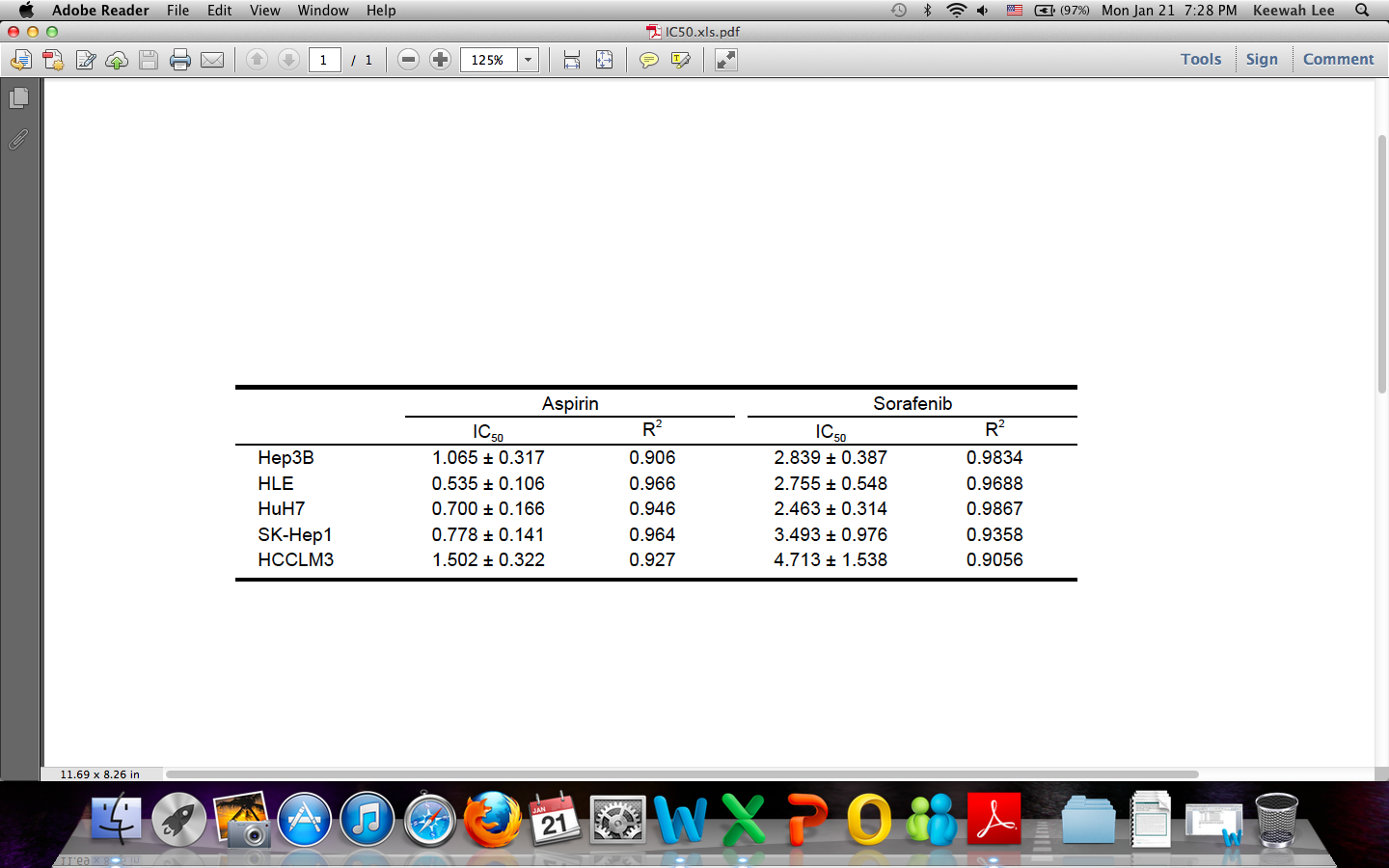
**Table S1. The IC_50_ for aspirin and sorafenib in 5 HCC cells.**

Abbreviation: IC_50_: The effective dose, which result in 50% of cell killing; Aspirin IC50: mg/ml, Sorafenib IC50: μg/ml. R^2^: Coefficient of determination, R^2^ values > 0.9 suggest that data are reliable and fit the statistical considerations.

###### Table S2. The combination index (CI) values of interactions between aspirin and sorafenib at varying concentration in Hep3B, Huh-7 and HCCLM3 cells after 24 hours treatment.


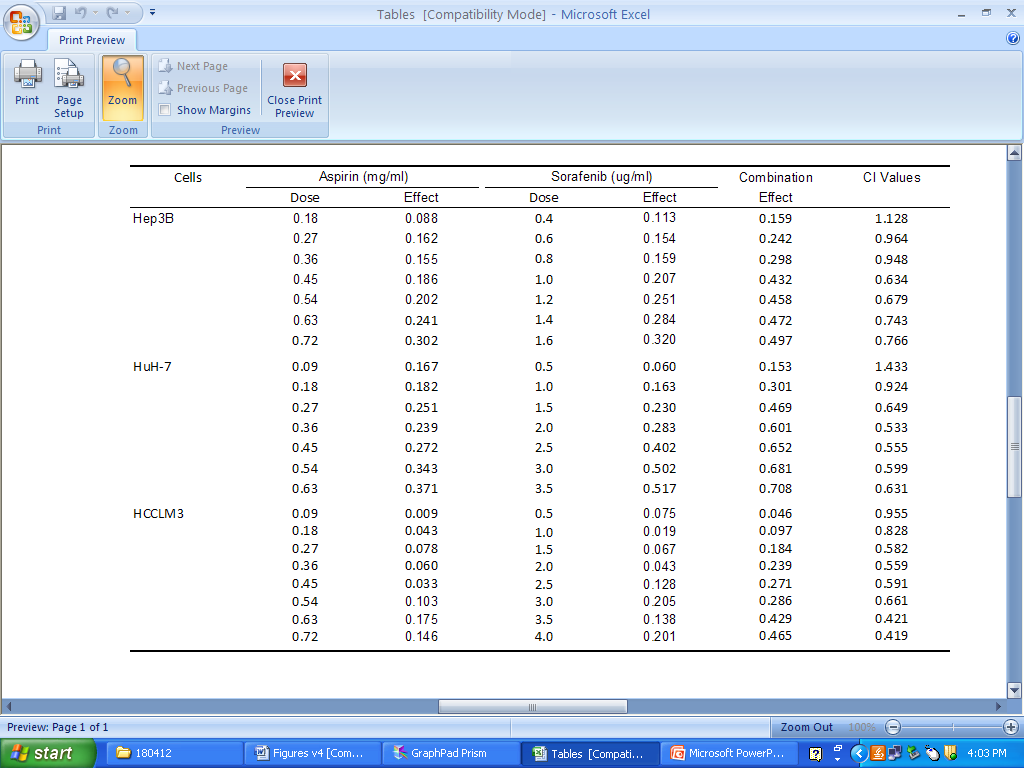


Abbreviation: CI values was generated when drugs were combined at a constant ratio (e.g. 0.45:1 for aspirin:sorafenib in Hep3B cells). CI values from 0.90 to 1.10 indicated additive interaction (±); CI from 0.85 to 0.90 indicates slight synergies (+); CI from 0.70 to 0.85 indicate moderate synergism (++); CI from 0.30 to 0.70 suggests synergism (+++); CI from 0.10 to 0.30 shows strong synergism (++++); and CI < 0.10 indicates very strong synergism.

**Table S3**. Aspirin and sorafenib combination inhibits upregulated genes in HCC but promotes expression of downregulated genes in HCC.

| **Gene Symbol** | **Hep3B-ASP vs. Con.** | **Hep3B-SOR vs. Con.** | **Hep3B-ASP+SOR vs. Con.** | **Huh7-ASP vs. Con.** | **Huh7-SOR vs. Con.** | **Huh7-ASP+SOR vs. Con.** | **T vs. MN** |
| --- | --- | --- | --- | --- | --- | --- | --- |
| **ACSL4** | -1.29706 | -1.47937 | -6.39695 | -1.06335 | -1.67284 | -10.4239 | 2.40228 |
| **ACSL4** | -1.28473 | -1.46983 | -6.00108 | -1.14622 | -1.42388 | -5.56147 | 2.27431 |
| PCDH17 | 1.34766 | -1.32833 | -1.97175 | 1.13176 | -2.93022 | -10.9332 | 2.0146 |
| FEN1 | -1.46112 | -1.52338 | -4.83398 | -1.05759 | -2.50436 | -3.0959 | 2.41459 |
| DUT | -1.13506 | -1.20691 | -3.71264 | 1.2974 | -2.64218 | -2.96411 | 2.10908 |
| HELLS | -1.14113 | -1.22684 | -2.88137 | -1.00783 | -1.87567 | -3.72565 | 2.79616 |
| GPC3 | -1.44364 | 1.27059 | -2.53904 | -1.12942 | 1.784 | -4.15296 | 2.76753 |
| HELLS | -1.11541 | -1.10252 | -2.69267 | -1.05169 | -1.94507 | -3.72835 | 3.88258 |
| FEN1 | -1.35467 | -1.39141 | -3.59821 | -1.06161 | -2.30414 | -2.70295 | 2.31188 |
| CHML | -1.39317 | -1.22249 | -3.70445 | -1.1291 | -1.29222 | -2.45194 | 2.10016 |
| ASPH | -1.20289 | -1.09509 | -3.0918 | -1.13839 | -1.14627 | -2.81995 | 2.04402 |
| TCF19 | 1.03062 | -1.21359 | -1.73802 | 1.11712 | -3.23605 | -4.27124 | 2.50271 |
| HELLS | -1.20279 | -1.26493 | -2.57967 | -1.08621 | -1.80032 | -2.79456 | 2.18042 |
| STMN1 | -1.02658 | -1.39112 | -3.08507 | -1.01187 | -1.76444 | -2.31845 | 2.63338 |
| LOC100506935 | 1.58472 | -1.02595 | -2.15198 | -1.18825 | -3.3542 | -3.31403 | 2.14841 |
| PCNA | -1.1957 | -1.27347 | -2.79769 | 1.01516 | -1.90492 | -2.47763 | 2.04812 |
| TRIP13 | -1.23279 | -1.25386 | -2.92692 | -1.04368 | -2.02562 | -2.34339 | 2.5064 |
| CDC6 | -1.176 | -1.11161 | -1.49003 | -1.15887 | -2.80605 | -4.58759 | 2.36411 |
| SPC25 | -1.18539 | -1.4836 | -3.31166 | -1.009 | -2.4221 | -2.03827 | 2.52511 |
| NQO1 | -1.69357 | 1.07804 | -4.50362 | 1.13146 | -1.26334 | -1.42919 | 2.27524 |
| PEG10 | -3.49393 | -2.1436 | -3.13593 | -1.06366 | -1.13529 | -2.04756 | 5.07939 |
| CDC7 | -1.0712 | -1.22303 | -2.52982 | 1.07543 | -1.66094 | -2.42654 | 2.68891 |
| WDR76 | 1.03898 | -1.16425 | -1.38963 | 1.14068 | -1.87464 | -4.0694 | 2.24012 |
| CENPW | -1.22224 | -1.34647 | -2.04609 | -1.12192 | -2.39944 | -2.75666 | 3.55891 |
| SOX2 | -3.13001 | -1.95673 | -5.88494 | 1.02033 | -1.01437 | 1.05696 | 2.0819 |
| CCNB1 | -1.21957 | -1.24049 | -3.03755 | 1.10908 | -1.32332 | -1.82994 | 3.71849 |
| CDC6 | -1.14523 | 1.10828 | -1.62602 | -1.15868 | -2.5702 | -3.32725 | 2.8648 |
| LYZ | -1.99757 | -1.27689 | -3.1939 | 1.04418 | -1.55156 | -1.68514 | 3.10608 |
| HIST1H3H | -1.1852 | -1.19184 | -1.20953 | -1.8173 | -2.09955 | -4.39388 | 2.93988 |
| LOC389834 | -1.26313 | -1.09797 | -3.80984 | 1.11314 | 1.20329 | -1.39416 | 2.02412 |
| CCL20 | -1.0045 | -1.15669 | -1.91642 | 1.0548 | -2.93353 | -2.74043 | 3.37423 |
| HOXA10 | 1.00242 | 1.08557 | -1.82494 | -1.73497 | -3.94211 | -2.85303 | 2.51762 |
| CTSC | -1.46892 | -1.10594 | -2.45147 | -1.02209 | -1.33628 | -2.01912 | 2.0086 |
| MCM3 | -1.17241 | -1.05109 | -2.24369 | -1.07095 | -1.56015 | -2.19178 | 2.33135 |
| KIAA1462 | -1.09355 | 1.0465 | -1.20928 | 1.13703 | -1.3495 | -3.8344 | 2.31137 |
| KIF14 | -1.19754 | -1.40487 | -1.532 | 1.05847 | -2.26327 | -3.02064 | 2.07403 |
| KIF11 | -1.1254 | -1.36289 | -1.89285 | -1.06102 | -2.14806 | -2.43869 | 2.91009 |
| COCH | 1.01341 | -1.06092 | -1.7104 | 1.06048 | -1.58996 | -2.65006 | 2.05202 |
| KIF14 | -1.0965 | -1.30809 | -1.42342 | 1.04537 | -2.29842 | -3.08908 | 3.23445 |
| BRIP1 | -1.10018 | -1.24136 | -1.57264 | -1.05771 | -1.73259 | -2.75521 | 2.34262 |
| HN1 | -1.03586 | -1.08431 | -1.69377 | -1.18318 | -2.46693 | -2.54337 | 2.24959 |
| MCM4 | -1.34082 | -1.22342 | -1.87022 | -1.03601 | -2.20928 | -2.24234 | 2.12201 |
| DUSP5 | 1.11129 | 1.00468 | 1.00536 | 1.22511 | -3.19515 | -4.21474 | -2.36187 |
| TDGF1 /1P3 | -1.85305 | -1.24399 | -2.9393 | -1.10242 | -1.64024 | -1.42238 | 2.0422 |
| HPGD | -1.21097 | -1.38448 | -1.95502 | -1.6258 | 1.16407 | -2.11687 | -3.84631 |
| EML6 | 1.4689 | -1.06743 | -1.38934 | 1.25212 | 2.30145 | -2.97358 | 2.59003 |
| ASPM | -1.0948 | -1.24065 | -2.00321 | 1.41514 | -1.29935 | -2.05399 | 3.47312 |
| TOMM40L | 1.08621 | -1.00495 | -1.1695 | 1.39623 | -1.36822 | -3.49689 | 2.52422 |
| RGS2 | -1.35524 | -1.5999 | -1.33811 | 1.07858 | -1.50903 | -3.03246 | -2.51333 |
| SFN | 1.47096 | 1.13074 | -1.25882 | -1.04687 | -3.38501 | -3.1788 | 2.61688 |
| MBNL2 | 1.1469 | 1.20263 | 2.47162 | 1.10885 | 1.46871 | 1.64898 | -3.04451 |
| ACSM3 | 1.0115 | 1.01067 | 1.00057 | 1.39064 | 2.0761 | 4.16202 | -2.67243 |
| EPB41L4B | 1.01399 | 1.23208 | 1.69735 | 1.41184 | 2.85233 | 2.50735 | -2.17491 |
| GADD45B | 1.00725 | -1.02038 | 3.19775 | -1.04744 | -1.0505 | 1.3785 | -2.1921 |
| WWC1 | 1.05866 | 1.09152 | 3.44353 | 1.08673 | 1.24803 | 1.28257 | -2.15121 |
| MT2A | 1.20809 | 1.04257 | 2.07237 | 1.13479 | 1.9031 | 2.14342 | -2.24082 |
| NAMPT | 1.04041 | 1.09153 | 1.57128 | 1.36919 | 2.48737 | 2.88949 | -2.75273 |
| MT1X | 1.07089 | 1.11868 | 1.89295 | 1.1344 | 2.164 | 2.4167 | -2.67108 |
| SERPINE1 | 2.44153 | -1.0166 | 8.02284 | 1.17524 | -3.14282 | -1.51536 | -2.23278 |
| CPEB3 | 1.17599 | -1.17181 | 2.81409 | 1.01131 | 1.11036 | 1.88261 | -3.2651 |
| SIK1 | 1.42148 | 1.43137 | 2.75467 | -1.02867 | 1.8437 | 1.92656 | -2.01517 |
| ITGA6 | 1.36526 | 1.84418 | 2.78552 | 1.22172 | 2.09673 | 1.91847 | 2.23971 |
| SH3YL1 | 1.45834 | 1.31174 | 1.78137 | 1.36047 | 2.77204 | 3.0435 | -2.13083 |
| DTNA | 1.03284 | 1.07806 | 2.00267 | 1.12464 | 1.82903 | 2.77257 | 2.53908 |
| MT1G | 1.13225 | 1.09797 | 2.04005 | 1.17974 | 2.3059 | 2.72522 | -3.71612 |
| BCO2 | 2.14434 | 1.35151 | 7.02635 | 1.13559 | -1.19476 | -1.24943 | -6.38953 |
| JUN | 1.41247 | 1.27147 | 2.18285 | 1.14168 | 1.12113 | 2.61274 | -2.09076 |
| SOCS2 | 1.40682 | 1.28649 | 4.14503 | -1.40574 | 1.18065 | 1.44449 | -3.26847 |
| LOC100505584 /// MT1E | 1.02337 | 1.11807 | 2.01757 | 1.21462 | 2.88591 | 3.12919 | -3.09581 |
| FOXP2 | 1.19738 | -1.03358 | 4.06549 | 1.14395 | 1.33199 | 1.56415 | -2.71203 |
| CYP4F2 /3 | -1.01078 | -1.01411 | -1.0346 | -1.03709 | 3.34007 | 6.86543 | -2.19847 |
| N4BP2L1 | 1.23389 | 1.04489 | 2.21378 | 1.41473 | 2.54101 | 3.00525 | -2.05376 |
| FOXP2 | 1.28973 | -1.11357 | 4.15819 | 1.09035 | 1.39914 | 1.60866 | -2.38193 |
| PPP1R3B | 1.00484 | -1.03688 | 6.35664 | 1.01435 | 1.11168 | 1.18991 | -2.40113 |
| SOCS2 | 2.25891 | 1.51439 | 4.57217 | -1.51365 | 1.05754 | 1.68204 | -3.56377 |
| LONRF1 | -1.00588 | 1.10358 | 2.84669 | 1.31057 | 2.6788 | 3.04417 | -2.17244 |
| GEM | -1.01884 | 1.01559 | -1.00178 | -1.56735 | 1.17832 | 9.68003 | -2.51644 |
| SORL1 | 1.50421 | 1.5815 | 4.13282 | 1.11533 | 1.59125 | 2.36011 | -2.34828 |
| KLF4 | -1.01855 | -1.02751 | 1.18946 | -1.37154 | 2.02618 | 8.46947 | -2.50478 |
| GABARAPL1 | 2.50184 | 1.21594 | 6.1619 | 1.05779 | -1.04137 | 1.87047 | -2.17921 |
| SORL1 | 1.3969 | 1.42917 | 5.07494 | 1.0705 | 1.43407 | 2.35476 | -2.42494 |
| SLC41A2 | 2.21945 | 1.26274 | 2.88279 | 1.02622 | 1.60103 | 4.2612 | -2.46427 |
| GABARAPL1/3 | 2.71626 | 1.24679 | 5.7452 | 1.02302 | -1.04153 | 2.14379 | -2.61434 |
| SORL1 | 1.02889 | 1.01257 | 11.1684 | -1.0153 | -1.00823 | 1.25999 | -4.04244 |
| NR3C2 | -1.05886 | -1.11242 | 3.71606 | 1.13648 | 1.84346 | 4.13955 | -2.07591 |
| SLC41A2 | 1.38851 | 1.06978 | 1.80054 | 1.71937 | 2.43752 | 9.07256 | -2.446 |
| PER1 | 1.15513 | 1.01372 | 4.03504 | 1.28747 | 1.45979 | 5.00646 | -2.37503 |
| TBX15 | 1.7638 | 1.66206 | 6.95985 | 1.21127 | 2.04001 | 3.3148 | -2.9389 |
| SLC41A2 | 1.38501 | -1.09031 | 2.36163 | 1.39602 | 3.28919 | 12.0362 | -2.09016 |
| **GADD45B** | 1.6282 | 1.22095 | 13.1874 | -1.03944 | 1.52449 | 3.3793 | -2.40305 |
| **GADD45B** | 1.61516 | 1.23551 | 13.2608 | -1.00817 | 1.56929 | 3.54994 | -2.62995 |
| **GADD45B** | 1.76517 | 1.18103 | 20.5843 | 1.23658 | 1.9813 | 5.56872 | -2.14714 |

**Supplementary Figure Legends**

**Figure S1** Annexin V-PE/7-AAD analysis of Hep3B (A) and HuH-7 (B) cells after treated 24, 48 and 72 hours with study agents. The quadrants images shown are representatives of 3 independent experiments and represent cells undergoing apoptosis.

**Figure S2.** (A) The panel of dysregulated genes that were upregulated in HCC human tissues and down-regulated only on treatment with aspirin and sorafenib combination (Supplementary Table S2) are mainly correlated with cell cycle, DNA damage and apoptosis related pathways by IPA analysis. (B) The signal network of GADD45 signalling by IPA analysis.

**Figure S3.** ACSL4 and GADD45B mediate the synergistic effects of combination aspirin and sorafenib. (A) The synergistic inhibition effect of combination aspirin and sorafenib was significantly strong in ACSL4+GADD45B- cell population than in ACSL4-GADD45B+ cell population. (B) The representative flow cytometry images showed that aspirin and sorafenib combination induces much more cell apoptosis in ACSL4+GADD45B- cell population than in ACSL4-GADD45B+ cell population isolated from HuH7 cells. (C) The percentage of cell apoptosis induced by aspirin and sorafenib combination in ACSL4+GADD45B- and ACSL4-GADD45B+ cell population isolated from Hep3B and HuH7 cells.

**Figure S4.** The association of ACSL4 and GADD45B expression with overall survival and recurrence free survival of HCC patients. The median expression value obtained for ACSL4 (A and C) and GADD45B (B and D) in the samples studied was chosen as the cut-off point for survival analysis using the Kaplan-Meier method.

**Figure S5.** The dotplot showed the expression of HTATIP2 and SDF1-α (CXCL12) in different datasets. (A, C, E) The expression of HTATIP2 did not significantly decreased in HCC in our dataset (A), TCGA dataset (C) and GSE14520 (E). (B, D, F) The expression of SDF1-α (CXCL12) was significantly decreased in HCC in our dataset (B), TCGA dataset (D) and GSE14520 (F).

**Figure S6**. The dotplot showed the expression of ACSL4 and GADD45B in different datasets. (A and B) The expression of ACSL4 was significantly increased in HCC in the TCGA dataset (A) and GSE14520 (B). (C and D) The expression of GADD45B was significantly decreased in HCC in the TCGA dataset (C) and GSE14520 (D).

**Supplementary Figures**

Figure S1


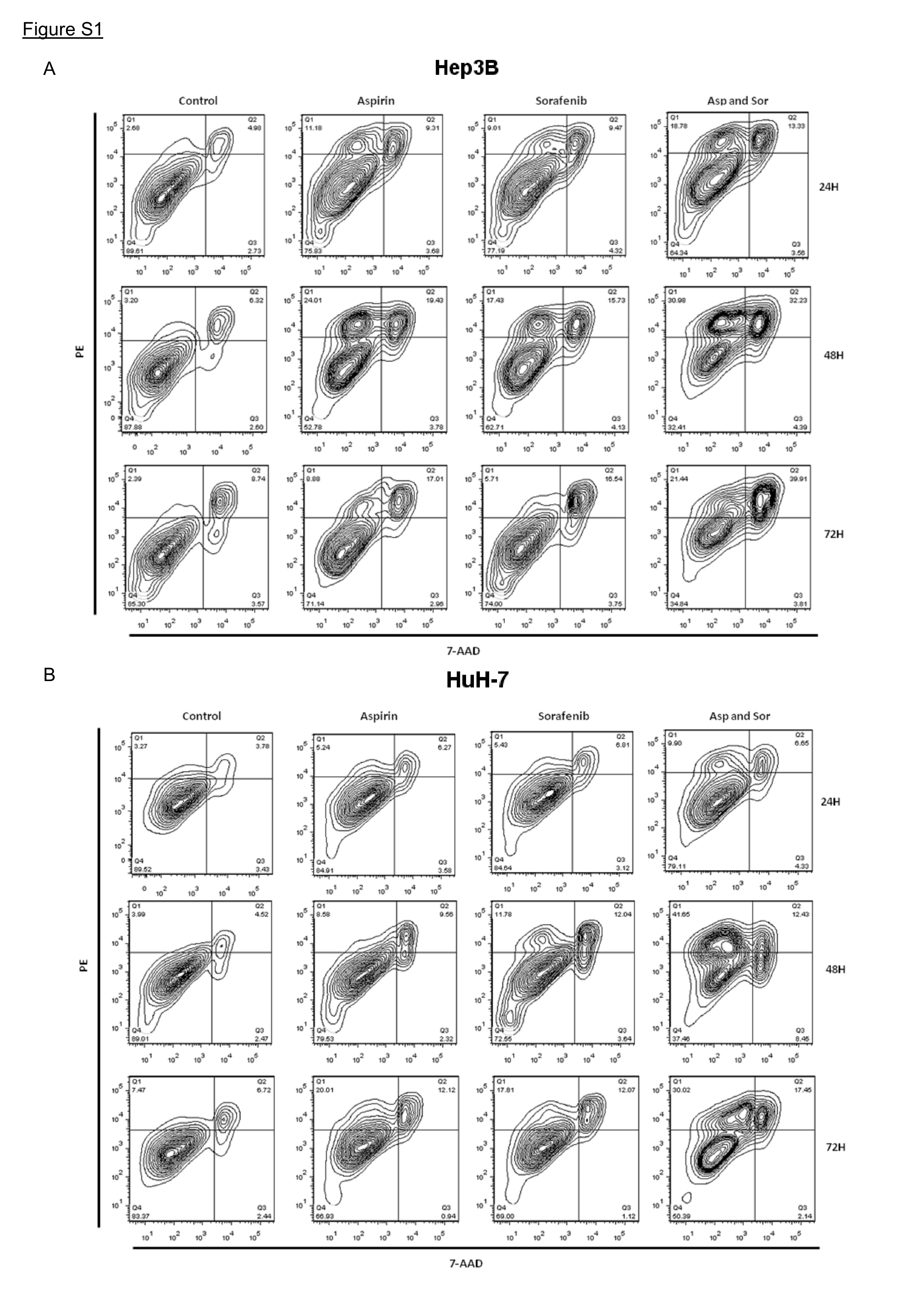


Figure S2


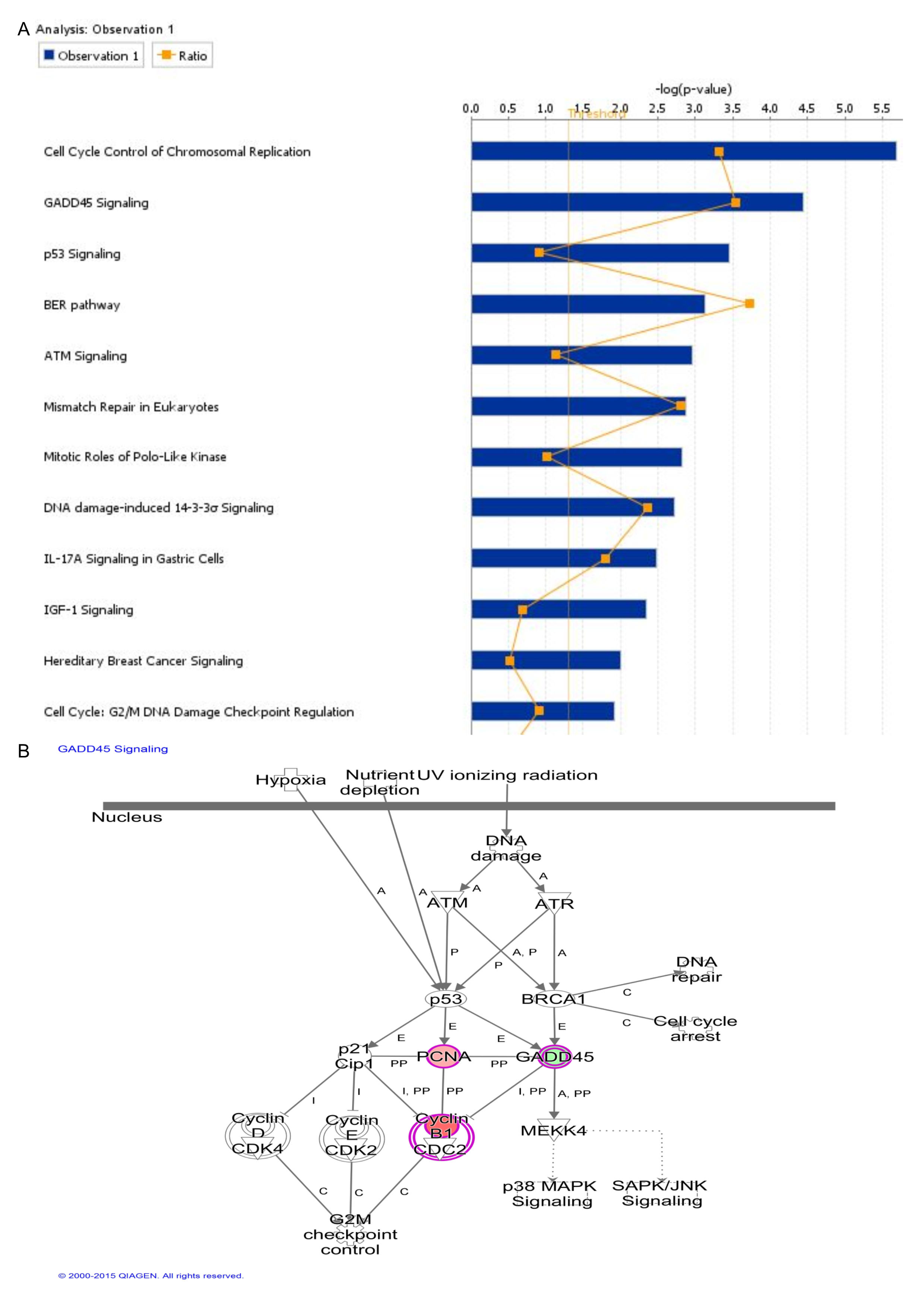


Figure S3


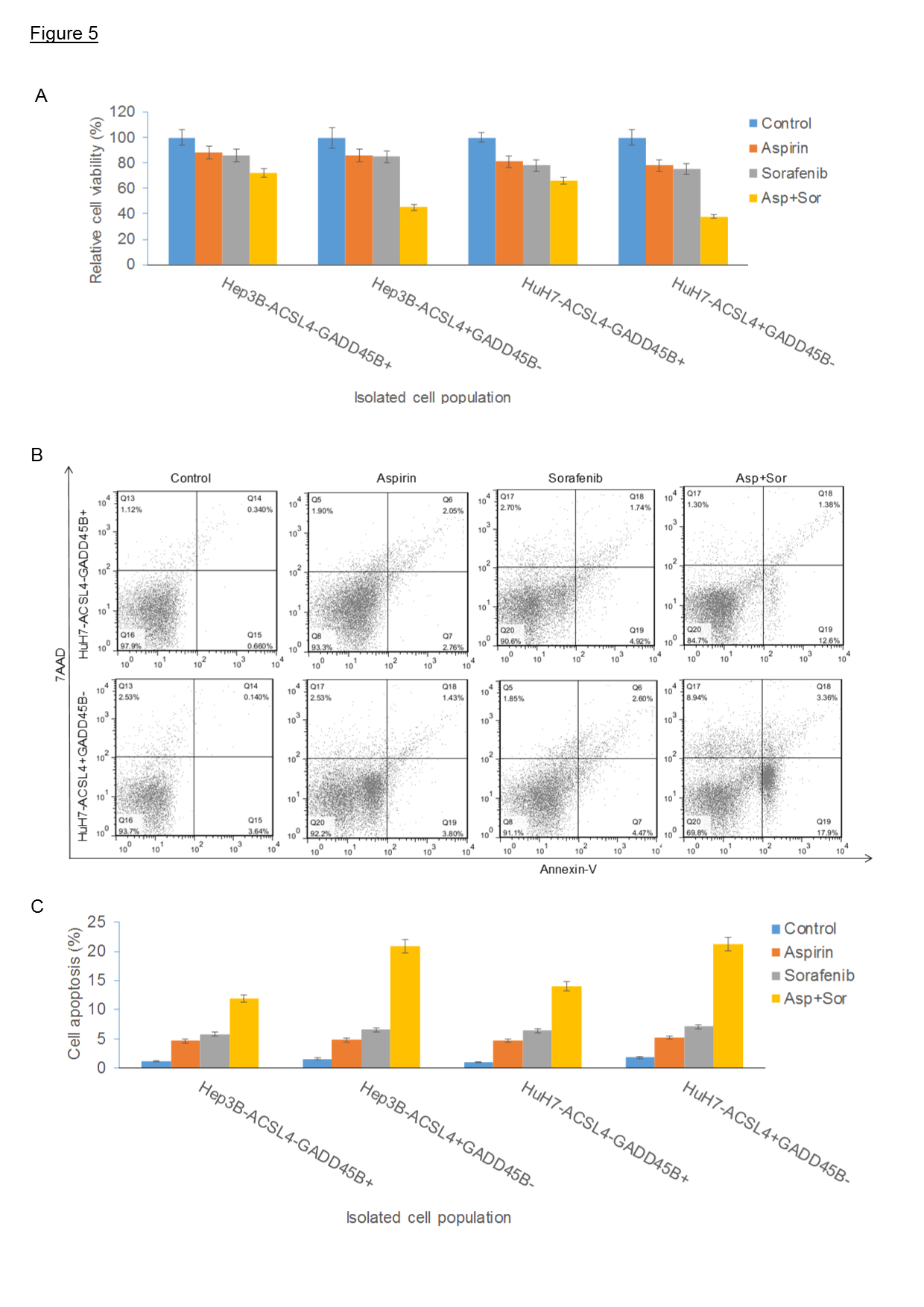


Figure S4


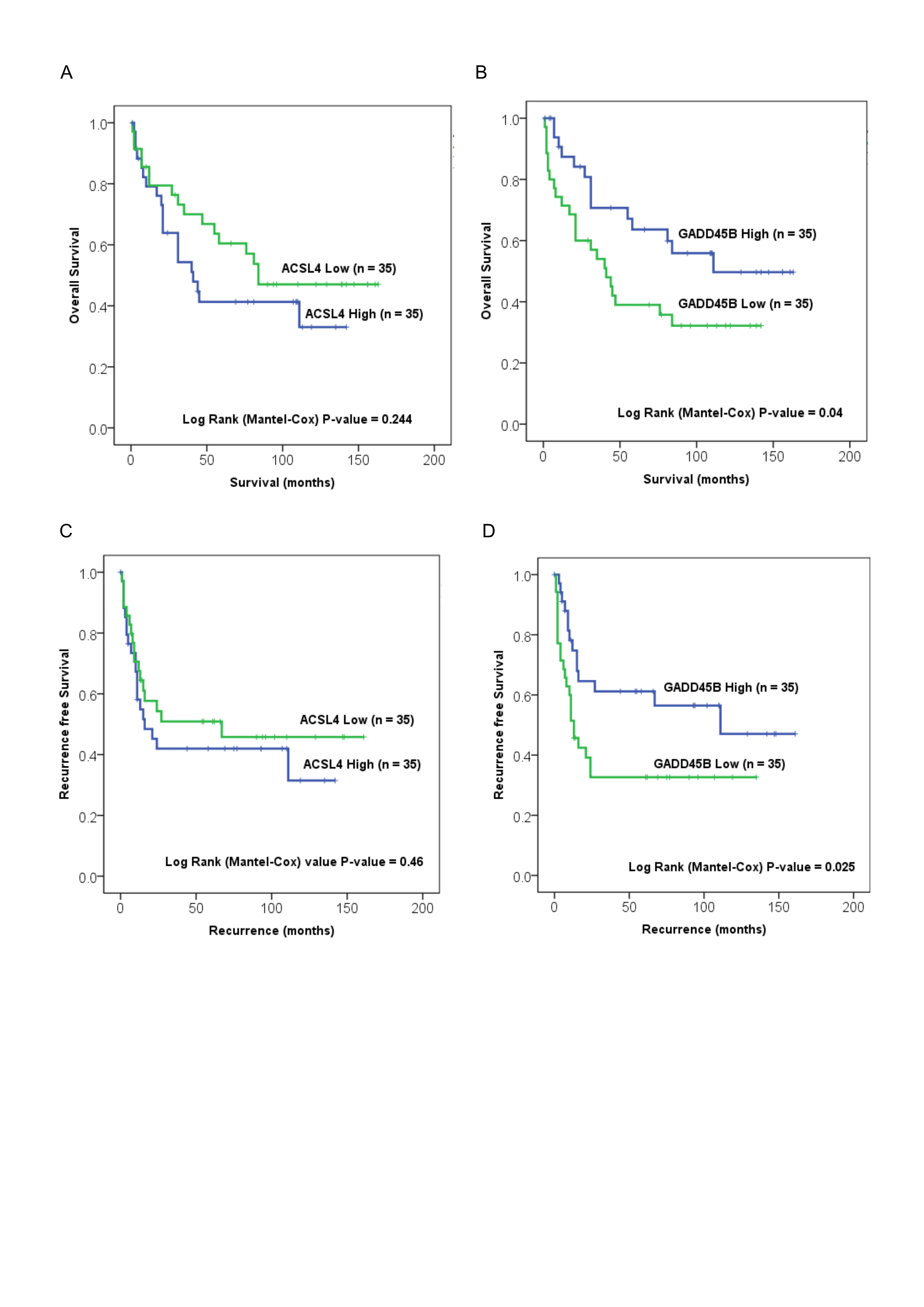


Figure S5


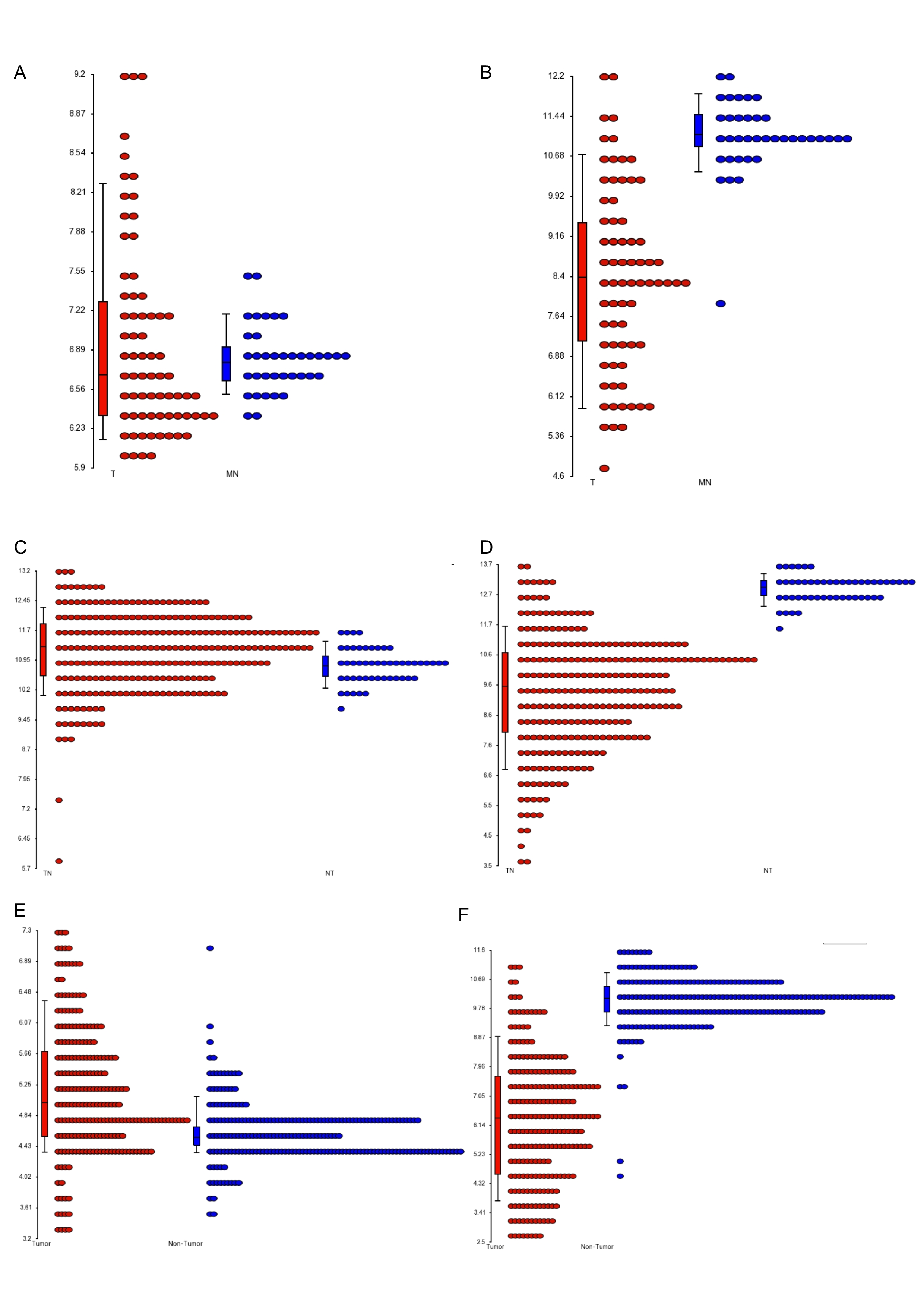


Figure S6


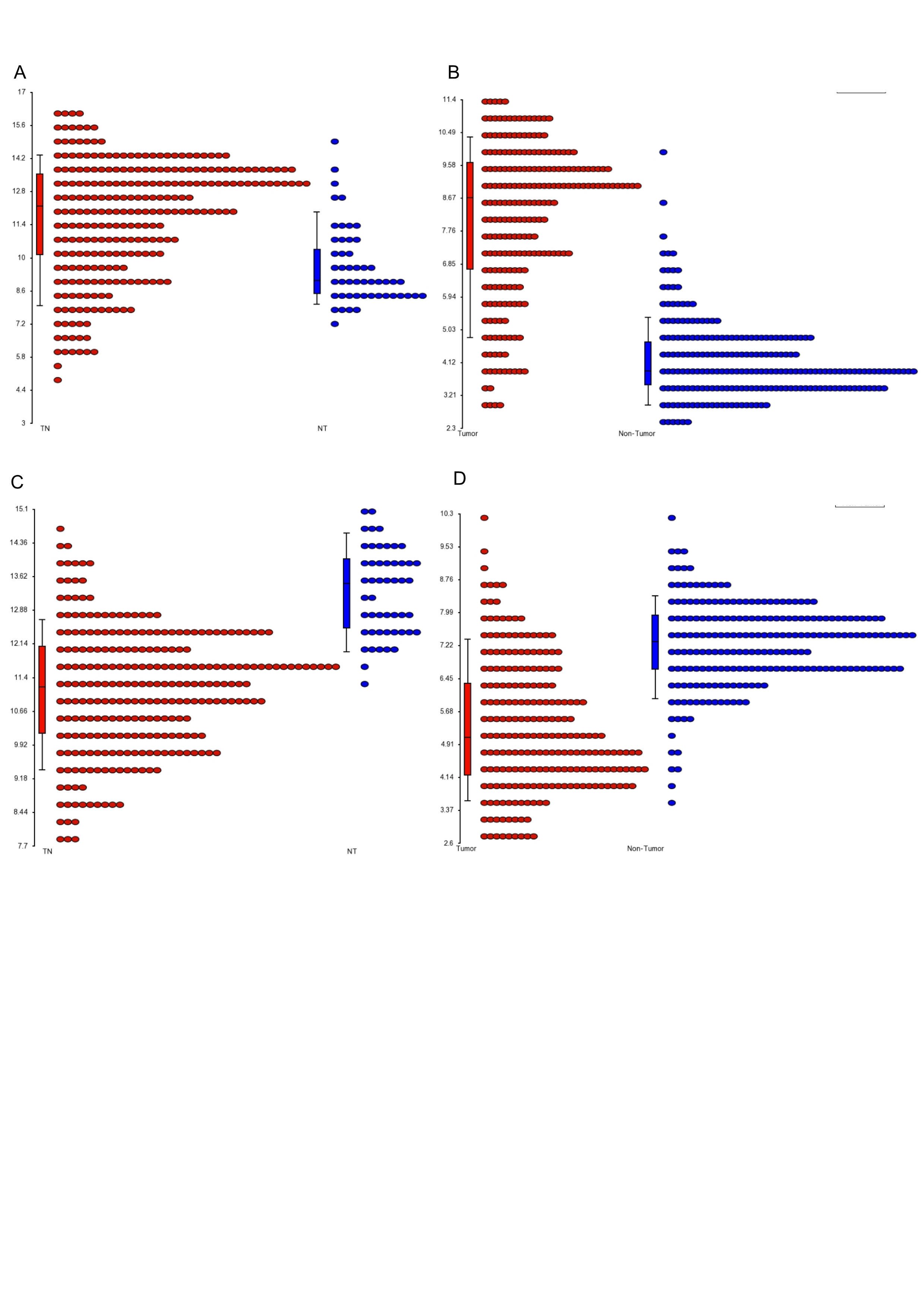

Supplement: Supplementary Information [file cddiscovery201758-s1.doc]
